# Hearing impairment and the risk of neurodegenerative dementia: A longitudinal follow-up study using a national sample cohort

**DOI:** 10.1038/s41598-018-33325-x

**Published:** 2018-10-15

**Authors:** So Young Kim, Jae-Sung Lim, Il Gyu Kong, Hyo Geun Choi

**Affiliations:** 1 Department of Otorhinolaryngology-Head & Neck Surgery, CHA Bundang Medical Center, CHA University, Seongnam, Korea; 2 Department of Neurology, Hallym University Sacred Heart Hospital, Anyang, Korea; 3 Department of Otorhinolaryngology-Head & Neck Surgery, Hallym University College of Medicine, Anyang, Korea

## Abstract

This study aimed to explore the risk of dementia in a middle- and older-aged population with severe or profound hearing impairments. Data were collected for the Korean National Health Insurance Service-National Sample Cohort from 2002 to 2013. Participants aged 40 or older were selected. The 4,432 severely hearing-impaired participants were matched 1:4 with 17,728 controls, and the 958 profoundly hearing-impaired participants were matched 1:4 with 3,832 controls who had not reported any hearing impairment. Age, sex, income, region of residence, hypertension, diabetes mellitus, and dyslipidemia histories were matched between hearing-impaired and control groups. The crude (simple) and adjusted (age, sex, income, region of residence, dementia, hypertension, diabetes mellitus, dyslipidemia, ischemic heart disease, cerebrovascular disease, and depression) hazard ratios (HRs) of hearing impairment on dementia were analyzed using Cox-proportional hazard models. The severe hearing impairment group showed an increased risk of dementia (adjusted HR = 1.17, 95% confidence interval [CI] = 1.04–1.31, P = 0.010). The profound hearing impairment group also showed an increased risk of dementia (adjusted HR = 1.51, 95% CI = 1.14–2.00, P = 0.004). Both severe and profound hearing impairments were associated with elevated the risk of dementia in middle- and older-aged individuals.

## Introduction

Dementia is a prevalent neurodegenerative disorder with a high economic and social burden. It has been reported that approximately 1% of individuals 60–70 years of age and 6–8% of individuals 85 years of age or older are diagnosed with dementia each year worldwide^1^. Because of the degenerative nature of the disease, the preclinical phase of dementia precedes the onset of dementia by more than 20 years^2^. Until now, the most effective approach to preventing dementia is controlling the modifiable risk factors for dementia. Among the various risk factors of dementia, regular physical activity and management of cardiovascular risk factors (obesity and smoking), and lifelong cognitive training were reported as modifiable risk factors^3^. In addition, hearing impairment has been proposed as a modifiable risk factor^4^.

Several previous studies have suggested a relation between dementia and age-related hearing decline^4^. However, few studies have investigated the risk of dementia in hearing-impaired subjects in wider age populations. Most previous studies have been conducted in populations 65 to 70 years of age and older^5–8^. For instance, a prospective cohort study reported that the moderate to severe hearing impairment was related to an increased risk of dementia in 70 and 79 years of age population^5^. On the other hands, a prospective study included younger subjects (ages 36–90 years) and found that severe hearing impairment increased the risk for all-cause dementia to 4.94 times that of the normal hearing control group^9^. However, this study had a small number of dementia subjects, with 37 cases of Alzheimer disease, and only 4 years of follow-up.

The hypothesis of the present study was that hearing impairment increases the risk of dementia in both middle- and older-aged subjects. To extend the findings of previous studies, this study used a large representative population and matched and/or adjusted several variables, including comorbidities. Furthermore, to explore the different impact of the hearing impairment on the risk of dementia according to severe or profound hearing impairment, the hearing impairment groups were divided into severely and profoundly hearing-impaired groups.

## Results

### Characteristics of Study Sample

**Figure 1:**
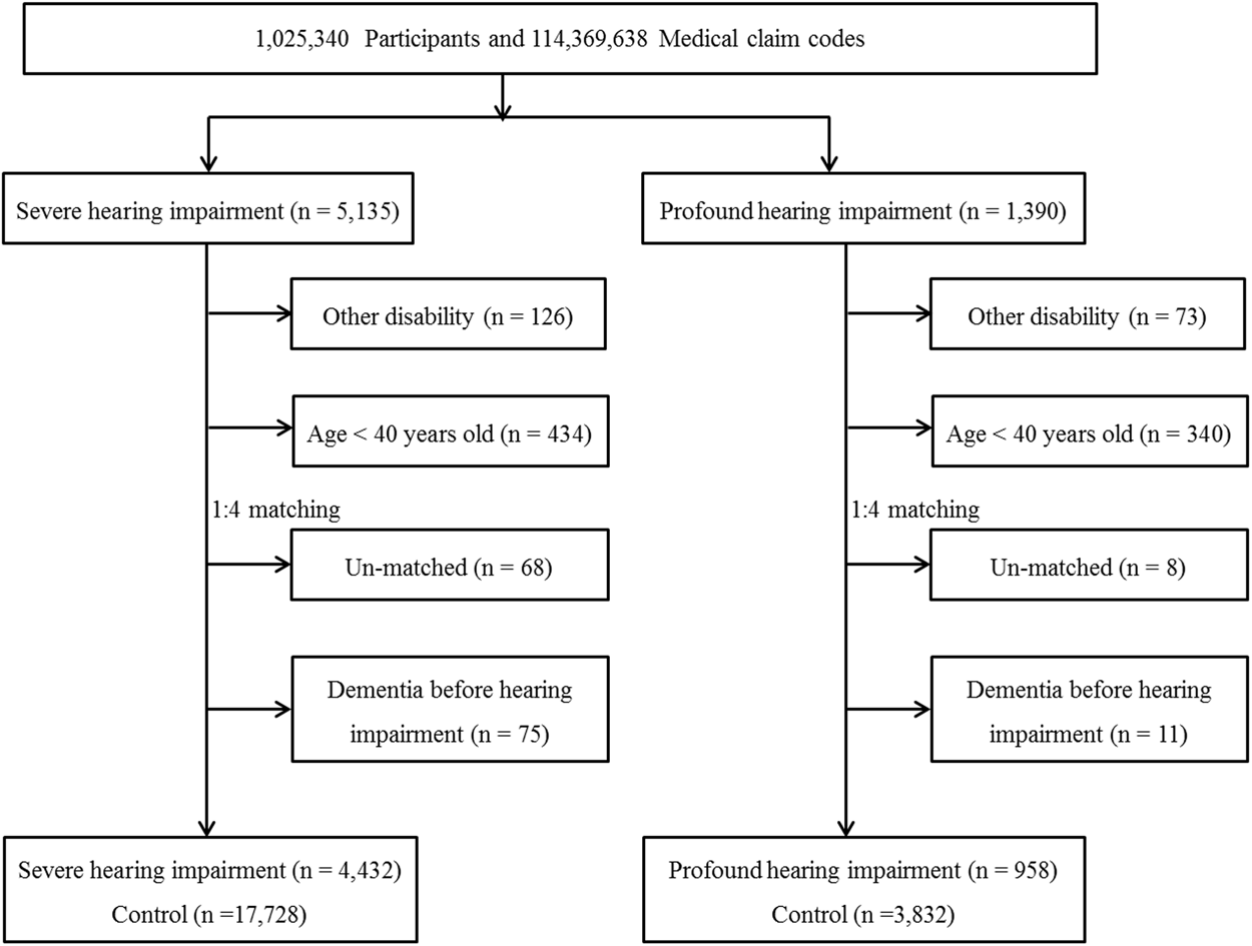
A schematic illustration of the participant selection process that was used in the present study. Out of a total of 1,025,340 participants, 5,135 severely hearing-impaired and 1,390 profoundly hearing-impaired participants were selected. The hearing-impaired participants were matched 1:4 with a control group that did not have a diagnosed hearing impairment. Finally, 4,432 severely hearing-impaired and 17,728 control participants and 958 profoundly hearing-impaired and 3,832 control participants were included.

The general characteristics of age, sex, income, region of residence, hypertension, diabetes mellitus, and dyslipidemia were matched between the hearing-impaired and control groups (Table 1 and Supplementary Table S1). Dementia was more frequent in the severely or profoundly hearing-impaired groups than in the control groups (8.3% vs. 6.6%, P < 0.001 for severe hearing impairment; 7.0% vs. 4.8%, P = 0.006 for profound hearing impairment) (Fig. 1A).

**Table 1:** General Characteristics of Participants.

Characteristics	Severe hearing impairment			Profound hearing impairment		
	Hearing impairment (n, %)	Control group (n, %)	P-value	Hearing impairment (n, %)	Control group (n, %)	P-value
Follow-up (months, mean [standard deviation])	88.61 (±40.85)	88.95 (±41.14)	1.000	124.05 (±31.35)	124.74 (±31.09)	1.000
Age (years old)			1.000			1.000
40–44	264 (6.0)	1,056 (6.0)		119 (12.4)	467 (12.4)	
45–49	327 (7.4)	1,308 (7.4)		107 (11.2)	428 (11.2)	
50–54	395 (8.9)	1,580 (8.9)		105 (11.0)	420 (11.0)	
55–59	536 (12.1)	2,144 (12.1)		125 (13.0)	500 (13.0)	
60–64	701 (15.8)	2,804 (15.8)		132 (13.8)	528 (13.8)	
65–69	754 (17.0)	3,016 (17.0)		126 (13.2)	504 (13.2)	
70–74	686 (15.5)	2,744 (15.5)		115 (12.0)	460 (12.0)	
75–79	487 (11.0)	1,948 (11.0)		77 (8.0)	308 (8.0)	
80–84	210 (4.7)	842 (4.7)		36 (3.8)	144 (3.8)	
85+	72 (1.6)	288 (1.6)		16 (1.7)	64 (1.7)	
Sex			1.000			1.000
Male	2,432 (54.9)	9,728 (54.9)		522 (54.5)	2,088 (54.5)	
Female	2,000 (45.1)	8,000 (45.1)		436 (45.5)	1,744 (45.5)	
Income			1.000			1.000
health aid (lowest)	357 (8.1)	1,428 (8.1)		198 (20.7)	792 (20.7)	
health insurance ≤10%	404 (9.1)	1,616 (9.1)		83 (8.7)	332 (8.7)	
11–20%	271 (6.1)	1,084 (6.1)		54 (5.6)	216 (5.6)	
21–30%	339 (7.6)	1,356 (7.6)		89 (9.3)	356 (9.3)	
31–40%	333 (7.5)	1,332 (7.5)		69 (7.2)	276 (7.2)	
41–50%	355 (8.0)	1,420 (8.0)		75 (7.8)	300 (7.8)	
51–60%	322 (7.3)	1,288 (7.3)		51 (5.3)	204 (5.3)	
61–70%	415 (9.4)	1,660 (9.4)		83 (8.7)	332 (8.7)	
71–80%	420 (9.5)	1,680 (9.5)		72 (7.5)	288 (7.5)	
81–90%	566 (12.8)	2,264 (12.8)		96 (10.0)	384 (10.0)	
≥91% (highest)	650 (14.7)	2,600 (14.7)		88 (9.2)	352 (9.2)	
Region of residence			1.000			1.000
Urban	1,778 (40.1)	7,112 (40.1)		334 (34.9)	1,336 (34.9)	
Rural	2,654 (59.9)	10,616 (59.9)		624 (65.1)	2,496 (65.1)	
Hypertension			1.000			1.000
Yes	2,683 (60.5)	10,732 (60.5)		462 (51.8)	1,848 (51.8)	
No	1,749 (39.5)	6,996 (39.5)		496 (48.2)	1,984 (48.2)	
Diabetes Mellitus			1.000			1.000
Yes	1,274 (28.7)	5,096 (28.7)		188 (19.6)	752 (19.6)	
No	3,158 (71.3)	12,632 (71.3)		770 (80.4)	3,080 (80.4)	
Dyslipidemia			1.000			1.000
Yes	1,259 (28.4)	5,063 (28.4)		202 (21.1)	808 (21.1)	
No	3,173 (71.6)	12,692 (71.6)		756 (78.9)	3,024 (78.9)	
Ischemic heart disease			0.060			0.562
Yes	503 (11.3)	1,840 (10.4)		83 (8.7)	310 (8.1)	
No	3,929 (88.7)	15,888 (89.6)		845 (91.3)	3,522 (91.9)	
Cerebrovascular disease			<0.001*			0.066
Yes	999 (22.5)	3,402 (19.2)		167 (17.4)	576 (15.0)	
No	3,433 (77.5)	14,326 (80.8)		791 (8.26)	3,256 (85.0)	
Depression			<0.001*			0.291
Yes	603 (13.6)	1,724 (9.7)		66 (6.9)	303 (7.9)	
No	3,829 (86.4)	16,004 (90.3)		892 (93.1)	3,529 (92.1)	

*Chi-square test, Significance at P < 0.05.

### Main Outcomes

Severe hearing impairment increased the risk of dementia (crude hazard ratio [HR] = 1.27, 95% confidence interval [CI] = 1.13–1.43, P < 0.001; adjusted HR = 1.17, 95% CI = 1.04–1.31, P = 0.010) (Table 2). The profoundly hearing-impaired group also demonstrated an increased risk of dementia (crude HR = 1.47, 95% CI = 1.11–1.94, P = 0.007; adjusted HR = 1.51, 95% CI = 1.14–2.00, P = 0.004) (Fig. 1B).

**Table 2:** Crude and adjusted hazard ratios (95% confidence interval) of hearing impairment for dementia.

Hazard ratios	Severe hearing impairment (n = 22,160)				Profound hearing impairment (n = 4,790)			
	Crude	P-value	Adjusted^†^	P-value	Crude	P-value	Adjusted^†^	P-value
Dementia	1.27 (1.13–1.43)	<0.001*	1.17 (1.04–1.31)	0.010*	1.47 (1.11–1.94)	0.007*	1.51 (1.14–2.00)	0.004*

*Cox-proportional hazard regression model in hearing impairment group compared to control, Significance at P < 0.05.

^†^Adjusted model for age, sex, income, region of residence, hypertension, diabetes mellitus, dyslipidemia, ischemic heart disease, cerebral stroke, and depression histories.

### Subgroup Analyses

We conducted the subgroup analyses according to age groups (Table 3). In the <65-year-old group, severe hearing impairment increased the risk of dementia only in the crude model (crude HR = 1.46, 95% CI = 1.06–2.11, P = 0.020; adjusted HR = 1.26, 95% CI = 0.92–1.74, P = 0.115). In this group, profound hearing impairment increased the risk of dementia (crude HR = 2.23, 95% CI = 1.19–4.19, P = 0.013; adjusted HR = 2.34, 95% CI = 1.22–4.49, P = 0.010). In ≥65-year-old group, severe hearing impairment increased the risk of dementia (crude HR = 1.25, 95% CI = 1.10–1.42, P = 0.001; adjusted HR = 1.16, 95% CI = 1.02–1.32, P = 0.021). In this group, profound hearing impairment increased the risk of dementia only in the adjusted model (crude HR = 1.34, 95% CI = 0.98–1.84, P = 0.063; adjusted HR = 1.38, 95% CI = 1.01–1.90, P = 0.043).

**Table 3:** Crude and adjusted hazard ratios (95% confidence interval) of hearing impairment for dementia in subgroup analysis according to age.

Hazard ratios	Severe hearing impairment				Profound hearing impairment			
	Crude	P-value	Adjusted^†^	P-value	Crude	P-value	Adjusted^†^	P-value
Dementia	Age < 65 years old (n = 11,115)				Age < 65 years old (n = 2,940)			
	1.46 (1.06–2.11)	0.020*	1.26 (0.92–1.74)	0.115	2.23 (1.19–4.19)	0.013*	2.34 (1.22–4.49)	0.010*
Dementia	Age ≥ 65 years old (n = 11,045)				Age ≥ 65 years old (n = 1,850)			
	1.25 (1.10–1.41)	0.001*	1.16 (1.02–1.32)	0.021*	1.34 (0.98–1.84)	0.063	1.38 (1.01–1.90)	0.043*

*Cox-proportional hazard regression model in hearing impairment group compared to control, Significance at P < 0.05.

^†^Adjusted model for age, sex, income, region of residence, hypertension, diabetes mellitus, dyslipidemia, ischemic heart disease, cerebral stroke, and depression histories.

## Discussion

The present study demonstrated that severe or profound hearing impairment elevated the risk of dementia in a ≥40 years old population. This study extended previous findings by including a middle-aged population instead of confining analyses to an older-aged population. As a result, middle-aged and older-aged adults with severe hearing impairments showed an increased risk of dementia. Moreover, profound hearing impairment showed higher HRs for dementia than for severe hearing impairment in this study.

Several plausible pathophysiologic mechanisms were suggested for the risk of dementia in hearing-impaired subjects. The deprivation of auditory sensory input might precipitate the decline of cognitive function in severely hearing-impaired subjects. A prospective cohort study of individuals aged 70–79 years demonstrated that hearing impairment was a risk factor for impairments of global cognitive and executive functions^10^. In a prior study, mice with moderate hearing impairment showed decreased learning and cognitive functions compared to normally hearing mice^11^. Although the present study investigated the new onset dementia after diagnosed as hearing impairment, the reverse causality might be possible between dementia and hearing impairment. Dementia or mild cognitive dysfunction was suggested to induce central auditory dysfunction^12,13^. Indirectly, the auditory deficit might make it difficult to communicate verbally, which may result in social isolation and contribute to cognitive impairment and subsequent dementia^14^. Emotional problems, including depression in hearing-impaired subjects, also contribute to the risk of dementia. The difficulties of hearing-impaired subjects in engaging in social activities have been suggested to induce psychological problems, including depression^15^. Loneliness and depressive symptoms could precipitate the risk of dementia^16^.

In addition to the causal relationship between hearing impairment and dementia, it was suggested that hearing impairment and dementia might coincide due to common neurodegenerative processes. Hearing impairment could be a harbinger of declining cognitive function. In particular, subjects with central auditory processing disorders could have central nervous dysfunction in non-auditory areas. Studies have suggested that degenerative changes in the central nervous system, including smaller brain volumes and white matter tract dysfunctions, may occur in hearing-impaired subjects^17^. White matter tract degeneration has been reported in early-stage dementia of the Alzheimer type^18^. Subjects with peripheral hearing impairment could progressively develop central hearing impairment at a later phase, with severe to profound hearing impairment^19^. Moreover, a recent electrophysiological study demonstrated that the central speech processing neural activity from the brainstem was impaired before other behavioral deficits occurred in mild cognitive impairment^20^. The common etiologic factors, including vascular compromise, inflammation, metabolic disturbance, and other hormonal factors between hearing impairment and dementia, might indicate an unraveling of shared pathophysiologic mechanisms. Future studies should delineate these links between hearing impairment and dementia.

This study demonstrated that the risk of dementia was higher in both middle-aged and older adult populations. The middle-aged adults demonstrated higher HR of dementia for profound hearing impairment than old adults in this study. The adverse impacts of profound hearing impairment could be stronger in middle-aged adults than in older adults, although the prevalence of hearing impairment is higher in older adults. It was reported that social isolation was higher in hearing-impaired adults aged 45–59 years than in hearing-impaired adults aged 60 years or older^14^. Because middle-aged adults have more opportunities to participate in social communication and often have jobs, they could be more affected by impaired verbal communication. In contrast, older adults tend to be more accommodating and often do not perceive their hearing impairment^21,22^. Thus, older adults might be more protected than middle-aged adults from the social isolation and emotional problems due to hearing impairment. In addition, subclinical cognitive impairments could initially manifest as a central auditory processing disorder in middle-aged adults as well as in older adults. Because early-onset Alzheimer disease in adults younger than 65 years of age has more aggressive features and atypical presentations compared to the more common late-onset Alzheimer disease, early detection and screening for severely hearing-impaired individuals might be crucial to relieving potential social and economic burdens^23^. Severe hearing impairment increased the risk of dementia in older adults but not in adults younger than 65 years of age in this study. The smaller number of severe hearing impairment and dementia patients in younger population than older adults might contribute to the less statistical power to demonstrate the risk of dementia in severe hearing impairment in adults younger than 65 years of age.

In the present study, profound hearing impairment showed a higher HR for dementia compared to severe hearing impairment. An animal experiment demonstrated that the degree of hearing impairment was correlated with neural degeneration in the hippocampus^24^. Despite a small sample size and a poorly adjusted or controlled study design, a previous study suggested that the degree of hearing impairment was related to the degree of cognitive deficit in both aided and unaided hearing-impaired subjects^25^. Some studies of participants with moderate or lower levels of hearing impairment have reported a weak association between hearing impairment and dementia or cognitive dysfunction. A longitudinal study did not show a relation between hearing impairment and mild cognitive impairment, which is probably because the study defined hearing impairment based on the whispered voice test rather than the pure tone audiometry (PTA)^12^. More deficits of auditory sensory deprivation might contribute to the elevated risk of dementia.

This study used representative nationwide population data that included middle- and older-aged individuals. By excluding young population, this study did not include the cases of congenital or childhood hearing impairments. Because the congenital hearing impairment could have different pathophysiology, such as genetic or infectious causes, with acquired hearing impairment, the impacts of hearing impairment on dementia had to be explored in acquired hearing impairment population. In the present study, severe hearing impairment was not confined to age-related hearing impairment, while most prior studies have focused on age-related hearing impairment. To minimize the effects of confounders, control groups were matched for age, sex, income, region of residence, hypertension, diabetes mellitus, and dyslipidemia. Furthermore, these matched variables and additional comorbidities of ischemic heart disease, cerebrovascular disease, and depression were adjusted. These comorbidities were adjusted but not matched with hearing impairment groups because the greater the number of matched variables, the greater the number of excluded subjects that would result. These excluded subjects might have elevated the possible selection bias. Thus, we did not match a comorbidity with relatively low incidence. Despite these strengths, there are some limitations of the present study. First, the severity of dementia could not be classified in this study. However, dementia was precisely diagnosed using the ICD-10 and twice or more patient treatment histories. The NHIS-NSC was validated for dementia in previous studies^26^. A previous research group analyzed the HRs for comorbid conditions, including disease of the eye and adnexa, endocrine and circulatory disease, musculoskeletal disease, respiratory disease, and nervous and mental disorders, in dementia patients using the NHIS-NSC data^26^. The Korea Dementia Comorbidity Index was derived from these HRs for comorbidities which as superior to other comorbidity indices for dementia^26^. In addition, the prevalence of dementia in this study was comparable to that of central dementia center of Korea (Supplementary File S1). Second, this study could not include participants with moderate or lower degrees of hearing impairment. Because the criteria for registered hearing-impaired subjects in Korea was a threshold of 60 dB or more in both ears, the audiometric thresholds could not be retrieved for subjects with mild to moderate hearing impairments. However, the fidelity of the diagnosis of hearing impairment was high in this study, through the use of PTA three times and an auditory brainstem response threshold test one time. Further studies examining a wider degree of hearing impairment and a sample of subjects categorized as having dementia will likely address the current limitations. Thirdly, It was possible that there might be changes of variables during the follow-up period in both study and control groups. However, these changes could not be accounted for analyses, because they will make models too complex to analyze in this study.

Severe hearing impairment increased the risk of dementia. Both middle-aged and older adults showed an elevated risk of dementia in the severely hearing-impaired group. Moreover, middle-aged group demonstrated higher HR of hearing impairment for dementia than older-aged group. Adults with profound hearing impairment showed a higher HR of dementia than adults with severe hearing impairment.

## Materials and Methods

### Study Population and Data Collection

The ethics committee of Hallym University (2014-I148) approved the use of these data. Written informed consent was exempted by the Institutional Review Board. All methods were performed in accordance with the guidelines and regulations of the ethic committee of Hallym University.

This national cohort study relied on data from the Korean National Health Insurance Service-National Sample Cohort (NHIS-NSC). The Korean National Health Insurance Service (NHIS) selects samples directly from the entire population database to prevent non-sampling errors. Approximately 2% of the samples (one million) was selected from the entire Korean population (50 million). These selected data can be classified at 1,476 levels (age [18 categories], sex [2 categories], and income level [41 categories]) using randomized stratified systematic sampling methods via proportional allocation to represent the entire population. After data selection, the appropriateness of the sample was verified by a previous study^27^. The details of the methods used to perform these procedures are provided by the National Health Insurance Sharing Service^28^. This cohort database included (i) personal information, (ii) health insurance claim codes (procedures and prescriptions), (iii) diagnostic codes using the International Classification of Diseases-10 (ICD-10), (iv) death records from the Korean National Statistical Office (using the Korean Standard Classification of Diseases), (v) socio-economic data (residence and income), and (vi) medical examination data for each participant from 2002 to 2013.

Because all Korean citizens are recognized by a 13-digit resident registration number from birth to death, exact population statistics can be determined using this database. It is mandatory for all Koreans to enroll in the NHIS. All Korean hospitals and clinics use the 13-digit resident registration number to register individual patients in the medical insurance system. Therefore, the risk of overlapping medical records is minimal, even if a patient moves from one location to another. Moreover, all medical treatments in Korea can be tracked, without exception, using the Health Insurance Review and Assessment (HIRA) system. In Korea, the notice of a death to an administrative entity is legally required before a funeral can be held.

### Hearing Measurements

Out of 1,025,340 cases with 114,369,638 medical claim codes, we included participants who were registered as a hearing-impaired person in the Ministry of Health and Welfare. These individuals were divided into 2 groups based on the degree of impairment: severe hearing impairment (hearing threshold ≥60 dB in both ears; ≥80 dB in one ear and ≥40 dB in one ear) and profound hearing impairment (hearing threshold ≥90 dB in both ears). In Korea, to be registered as a hearing-impaired person, individuals must be checked 3 times using a pure tone audiometry test (PTA) and 1 time using an auditory brainstem response. The average hearing threshold of PTA was calculated as follows: (500 Hz + 2 * 1000 Hz + 2 * 2000 Hz + 4000 Hz)/6.

### Participant Selection

**Figure 2:**
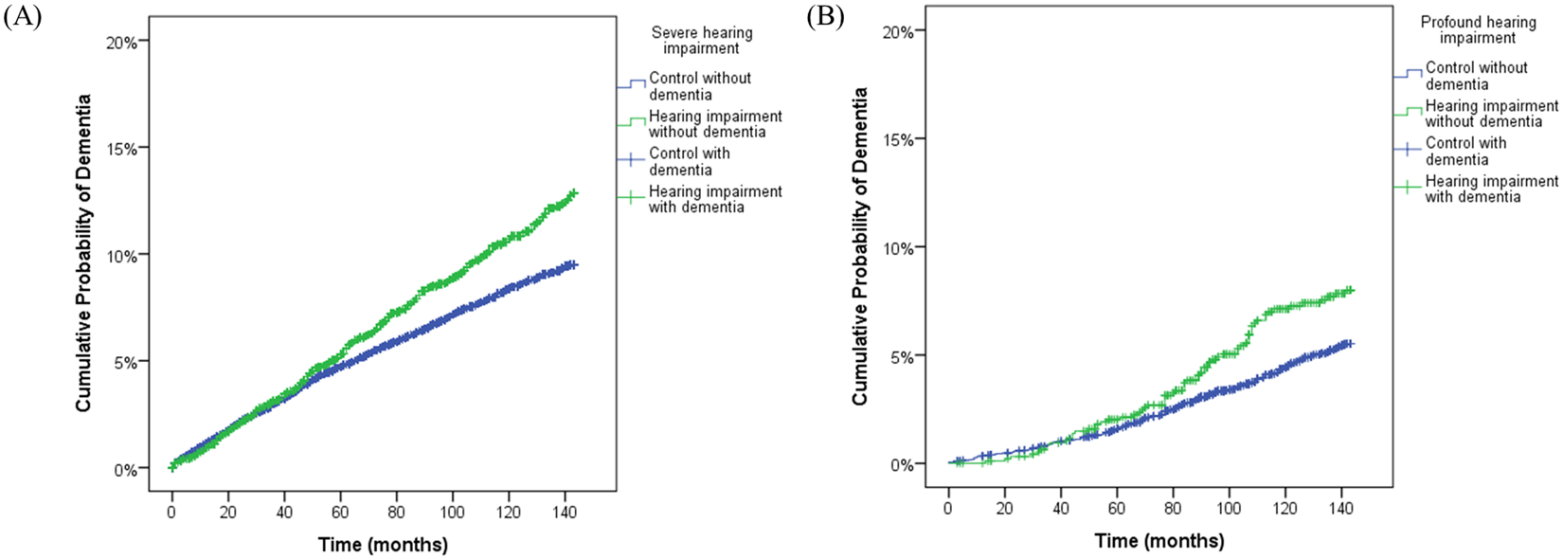
The Kaplan-Meier survival analysis showed that both the groups with severe (**A**) and profound (**B**) hearing impairment demonstrated a higher cumulative rate of dementia than the control groups

A total of 5,135 severely hearing-impaired and 1,390 profoundly hearing-impaired participants were selected. Of these participants, those who were registered with other disabilities (physical disability, brain lesion disorder, visual loss, mental retardation, psychiatric disorder, kidney disorder, and others) in the Ministry of Health and Welfare were excluded (severe hearing impairment, n = 126; profound hearing impairment, n = 73). We also excluded participants under 40 years of age in 2002 (severe hearing impairment, n = 434; profound hearing impairment, n = 340). The hearing-impaired participants were matched 1:4 with participants (control group) who had never been diagnosed with a hearing impairment or other disability from 2002 through 2013. The matches were processed for age, group, sex, income group, region of residence, hypertension, diabetes mellitus, and dyslipidemia histories. However, they were not matched for ischemic heart disease, cerebral stroke, and depression because strict matching increases the number of excluded study participants due to a lack of control participants. After matching, we analyzed the participants’ histories of dementia in both the hearing impairment and control groups. To prevent selection bias when selecting the matched participants, the control group participants were sorted using a random number order, and they were then selected from top to bottom. It was assumed that the matched control participants were involved at the same time of each matched hearing-impaired participant. Therefore, members of the control group who had died before the time of involvement of the matched hearing-impaired participants were excluded. The hearing-impaired participants for whom we could not identify enough matching participants were excluded (severe hearing impairment, n = 68; profound hearing impairment, n = 8). The participants who had dementia before being diagnosed with a hearing impairment were excluded (severe hearing impairment, n = 75; profound hearing impairment, n = 11). Finally, a 1:4 matching resulted in the inclusion of 4,432 severely hearing-impaired participants and 17,728 corresponding control participants and 958 profoundly hearing-impaired participants and 3,832 corresponding control participants (Fig. 2).

### Definition of Dementia

Dementia was categorized if the participants were diagnosed with Alzheimer’s disease (G30) or dementia in Alzheimer’s disease (F00). For the accuracy of diagnosis, we selected these options only if the participants were treated ≥2 times. We have described the reliability of the diagnosis of dementia in the supplement (Supplementary File S1).

### Variables

The age groups were classified using 5-year intervals (i.e., 40–44, 45–49, 50–54…, and 85+ years old). A total of 10 age groups were designated. The income groups were initially divided into 41 classes (one health aid class, 20 self-employment health insurance classes, and 20 employment health insurance classes). These groups were re-categorized into 11 classes (one health aid class, 10 health insurance classes). The health insurance population was grouped according to 10 deciles. This re-categorization was performed by NIHS to de-identification of participants. Regions of residence were divided into 16 areas according to administrative districts. These regions were regrouped into urban (Seoul, Busan, Daegu, Incheon, Gwangju, Daejeon, and Ulsan) and rural (Gyeonggi, Gangwon, Chungcheongbuk, Chungcheongnam, Jeollabuk, Jeollanam, Gyeongsangbuk, Gyeongsangnam, and Jeju) areas.

The past medical histories of participants were evaluated using ICD-10 codes as previous studies^29,30^. For the accuracy of diagnosis, hypertension (I10 and I15), diabetes (E10–E14), and hyperlipidemia (E78) were selected if participants were treated ≥2 times. Ischemic heart disease (I24 and I25) and cerebrovascular disease (I60–I66) were selected if participants were treated ≥1 time. Depression was defined using the ICD-10 codes F31 (bipolar affective disorder) through F39 (unspecified mood disorder), as specified by a psychiatrist from 2002 through 2013.

### Statistical Analyses

Chi-square tests were used to compare the general characteristics between the hearing-impaired group and the control group.

To analyze the HR of hearing impairment on dementia, Cox-proportional hazard models were used. In this analysis, crude (simple) and adjusted (age, sex, income, region of residence, dementia, hypertension, diabetes mellitus, dyslipidemia, ischemic heart disease, cerebrovascular disease, and depression) models were used. In addition, 95% CIs were calculated. To analyze the cumulative probability of dementia in the hearing-impaired and control groups, Kaplan-Meier survival analysis was conducted and Kaplan-Meier plot was provided. Cox proportional hazards model test was performed. For the subgroup analysis, we divided the participants by age (<65 years old and ≥65 years old).

Two-tailed analyses were conducted, and P-values less than 0.05 were considered significant. The results were statistically analyzed using SPSS v. 21.0 (IBM, Armonk, NY, USA).

## Electronic supplementary material

Supplementary Table S1
