# Supplementary material for: Hearing impairment and the risk of neurodegenerative dementia: A longitudinal follow-up study using a national sample cohort
Source: Sci Rep. 2018 Oct 15;8:15266. doi: 10.1038/s41598-018-33325-x (PMC6189102; doi:10.1038/s41598-018-33325-x)
Supplement: Supplementary file 1 — Supplementary Table S1 [file 41598_2018_33325_MOESM1_ESM.docx]

**Hearing impairment and the risk of neurodegenerative dementia: A longitudinal follow-up study using a national sample cohort**

So Young Kim, MD^1^, Jae-Sung Lim, MD^2^, Il Gyu Kong, MD^3^, Hyo Geun Choi, MD^3^

^1^Department of Otorhinolaryngology-Head & Neck Surgery, CHA Bundang Medical Center, CHA University, Seongnam, Korea

^2^Department of Neurology, Hallym University Sacred Heart Hospital, Anyang, Korea

^3^Department of Otorhinolaryngology-Head & Neck Surgery, Hallym University College of Medicine, Anyang, Korea

*Correspondence: [pupen@naver.com](mailto:pupen@naver.com)

**Supplementary Table S1** The prevalence of dementia in severe and profound hearing impairment groups

| Characteristics | | Severe hearing impairment (n, %) | Profound hearing impairment (n, %) |
| --- | --- | --- | --- |
| Age (years old) | |  |  |
|  | 40-44 | 2 (0.2) | 3 (0.5) |
|  | 45-49 | 2 (0.1) | 2 (0.4) |
|  | 50-54 | 9 (0.5) | 3 (0.6) |
|  | 55-59 | 46 (1.7) | 10 (1.6) |
|  | 60-64 | 132 (3.8) | 24 (3.6) |
|  | 65-69 | 278 (7.4) | 49 (7.8) |
|  | 70-74 | 444 (12.9) | 70 (12.2) |
|  | 75-79 | 392 (16.1) | 54 (14.0) |
|  | 80-84 | 180 (17.1) | 30 (16.7) |
|  | 85+ | 49 (13.6) | 6 (7.5) |
| Sex | |  |  |
|  | Male | 613 (5.0) | 101 (3.9) |
|  | Female | 921 (9.2) | 150 (6.9) |
| Income | |  |  |
|  | 1 (lowest) | 184 (10.3) | 44 (4.4) |
|  | 2 | 136 (6.7) | 28 (6.7) |
|  | 3 | 75 (5.5) | 4 (1.5) |
|  | 4 | 100 (5.9) | 13 (2.9) |
|  | 5 | 97 (5.8) | 14 (4.1) |
|  | 6 | 88 (5.0) | 12 (3.2) |
|  | 7 | 82 (5.1) | 15 (5.9) |
|  | 8 | 102 (4.9) | 26 (6.3) |
|  | 9 | 157 (7.5) | 19 (5.3) |
|  | 10 | 211 (7.5) | 30 (6.3) |
|  | 11 (highest) | 302 (9.3) | 46 (10.5) |
| Region of residence | |  |  |
|  | Urban | 558 (6.3) | 90 (5.4) |
|  | Rural | 976 (7.4) | 161 (5.2) |
| Hypertension | |  |  |
|  | Yes | 1,193 (8.9) | 184 (8.0) |
|  | No | 341 (3.9) | 67 (2.7) |
| Diabetes Mellitus | |  |  |
|  | Yes | 535 (8.4) | 76 (8.1) |
|  | No | 999 (6.3) | 175 (4.5) |
| Dyslipidemia | |  |  |
|  | Yes | 394 (6.3) | 75 (7.4) |
|  | No | 1,140 (7.2) | 176 (4.7) |
| Ischemic heart disease | |  |  |
|  | Yes | 2243 (10.4) | 35 (8.9) |
|  | No | 1,291 (6.5) | 216 (4.9) |
| Cerebrovascular disease | |  |  |
|  | Yes | 711 (16.2) | 103 (13.9) |
|  | No | 823 (4.6) | 148 (3.7) |
| Depression | |  |  |
|  | Yes | 387 (16.6) | 56 (15.2) |
|  | No | 1,147 (5.8) | 195 (4.4) |

* Chi-square test, Significance at P < 0.05

**Supplementary File S1**. **Description of diagnosis of dementia**

Dementia was categorized if the participants were diagnosed Alzheimer's disease (G30) or Dementia in Alzheimer's disease (F00). We selected if the participants were treated ≥ 2 times.

In this national sample cohort, 123,025 participants were ≥ 65 years old in 2012 year. Among them, 9,740 (7.9%) of participants were categorized as dementia according to our methods (5.4% [n =2,758] in male; 9.7% [n= 6,982] in female).

We could compare these results of central dementia center of Korea ([www.nid.or.kr](http://www.nid.or.kr)) which is controlled by Ministry of Health and Welfare of Korea. The earliest data was 2012 year, and it was available in ≥ 65 years old. According to their data, the prevalence of dementia (Alzheimer's disease, and others) except vascular dementia were 7.63% (4.47% in male; 9.85% in female).
